# Remarkable Effect of Gefitinib Retreatment in a Lung Cancer Patient With Lepidic Predominat Adenocarcinoma who had Experienced Favorable Results From Initial Treatment With Gefitinib: A Case Report

**DOI:** 10.4021/jocmr816e

**Published:** 2012-05-15

**Authors:** Su Jin Lee, Ho Sung Lee, Jae Sung Choi, Ju Ock Na, Ki Hyun Seo, Mi Hye Oh, Sung Shick Jou

**Affiliations:** a Department of Internal medicine, Collage of Medicine, Soonchunhyang University, Cheonan, Republic of Korea; b Diagnostic Pathology, Collage of Medicine, Soonchunhyang University, Cheonan, Republic of Korea; c Diagnostic Radiology, Collage of Medicine, Soonchunhyang University, Cheonan, Republic of Korea

**Keywords:** Gefitinib, Retreatment, Lepidic predominant adenocarcinoma

## Abstract

Gefitnib is an oral agent of epidermal growth factor receptor tyrosine kinase inhibitor, and it has a certain efficacy against non-small cell lung cancer. There are some reports that the non-small cell lung cancer patients who experienced disease progression after responding to gefitinib were again sensitive to re-administration of gefitinib following temporary cessation of gefitinib. This is the case report showing a remarkable effect of gefitinib re-treatment in a patient with metastatic invasive adenocarinoma who had experienced favorable results from the initial treatment with gefitinib.

## Introduction

The over-activation of epidermal growth factor receptor (EGFR) is involved in proliferation and survival of cancer cells. Gefitinib is an oral agent of epidermal growth factor receptor tyrosine kinase inhibitor, and it has a certain radiographic and symptomatic efficacy against non-small cell lung cancer. It was approved in Japan in July 2002 for the treatment of patients with non-small cell lung cancer [1], especially in females, those with an adenocarcinoma histology, those who have never smoked, and patients with a good performance status [1, 2]. There are some reports of non-small cell lung cancer patients who experienced disease progression after initially responding to gefitinib, who were again sensitive to re-administration of gefitinib following temporary cessation of gefitinib [3-6]. It is of interest whether re-challenging with gefitinib after treatment of a recurrence site contributes to the patient’s survival by achieving partial response or by maintaining the disease stable for a long time. In the present study, we report on a case of a remarkable effect of gefitinib re-treatment in a lung cancer patient with lepidic predominant adenocarcinoma who had experienced favorable results from initial treatment with gefitinib.

## Case Report

**Figure 1:**
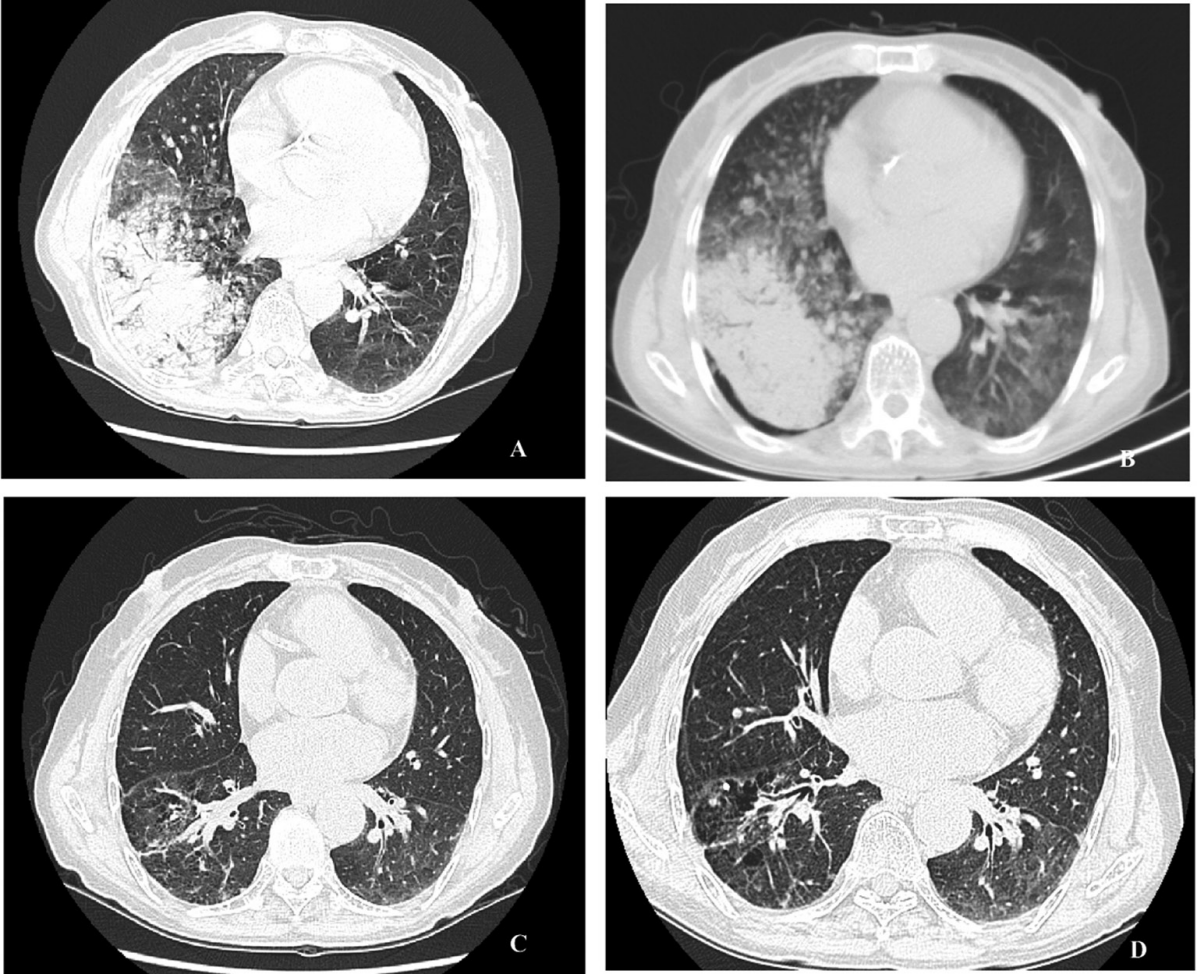
A: Initial chest CT scan, a consolidative mass in RML and RLL with multiple hematogenous metastatic nodules in BUL. B: Chest CT scan before treatment with 250 mg gefitinib, more increased consolidative mass in the right lung field. C: Chest CT scan 6 months after treatment with 250 mg gefitinib, much improved consolidative mass in the right lung and markedly improved hematogenous metastasis in BUL. D: Chest CT scan 13 month after treatment with 250 mg gefitinib, no interval change compared with the previous CT scan.

**Figure 2:**
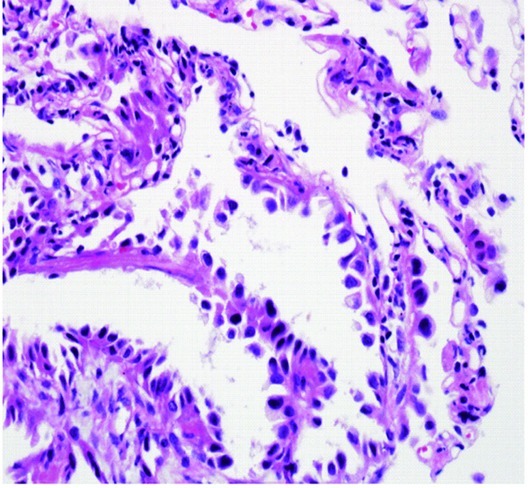
Photomicroscopic finding of lepidic predominant adenocarcinoma (H&E, × 400).

**Figure 3:**
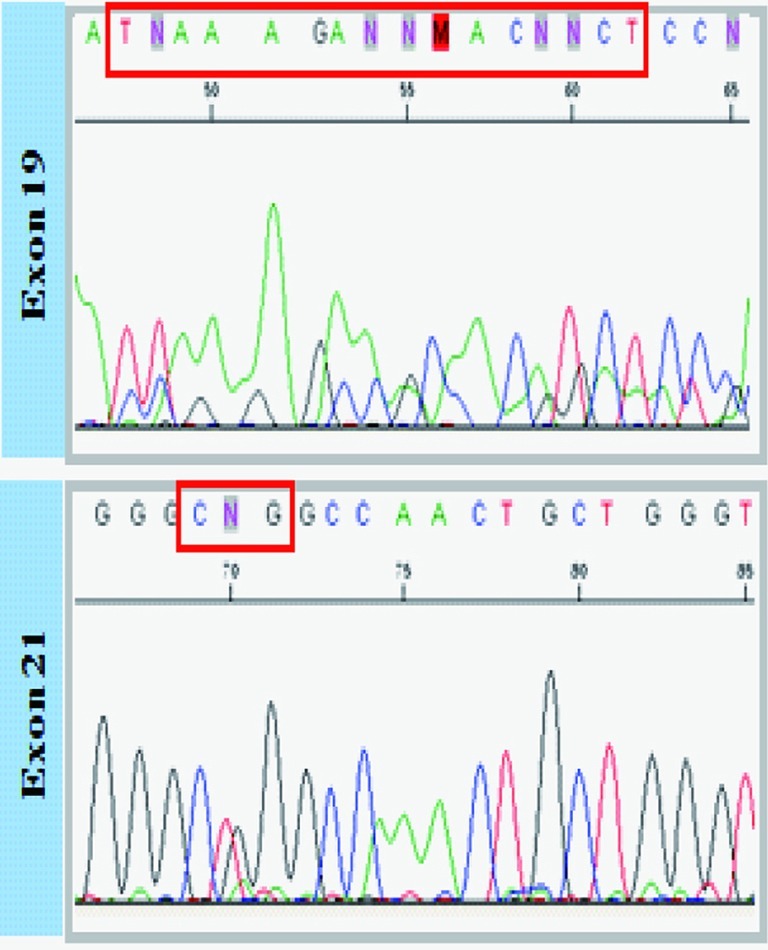
EGFR gene analysis.

**Figure 4:**
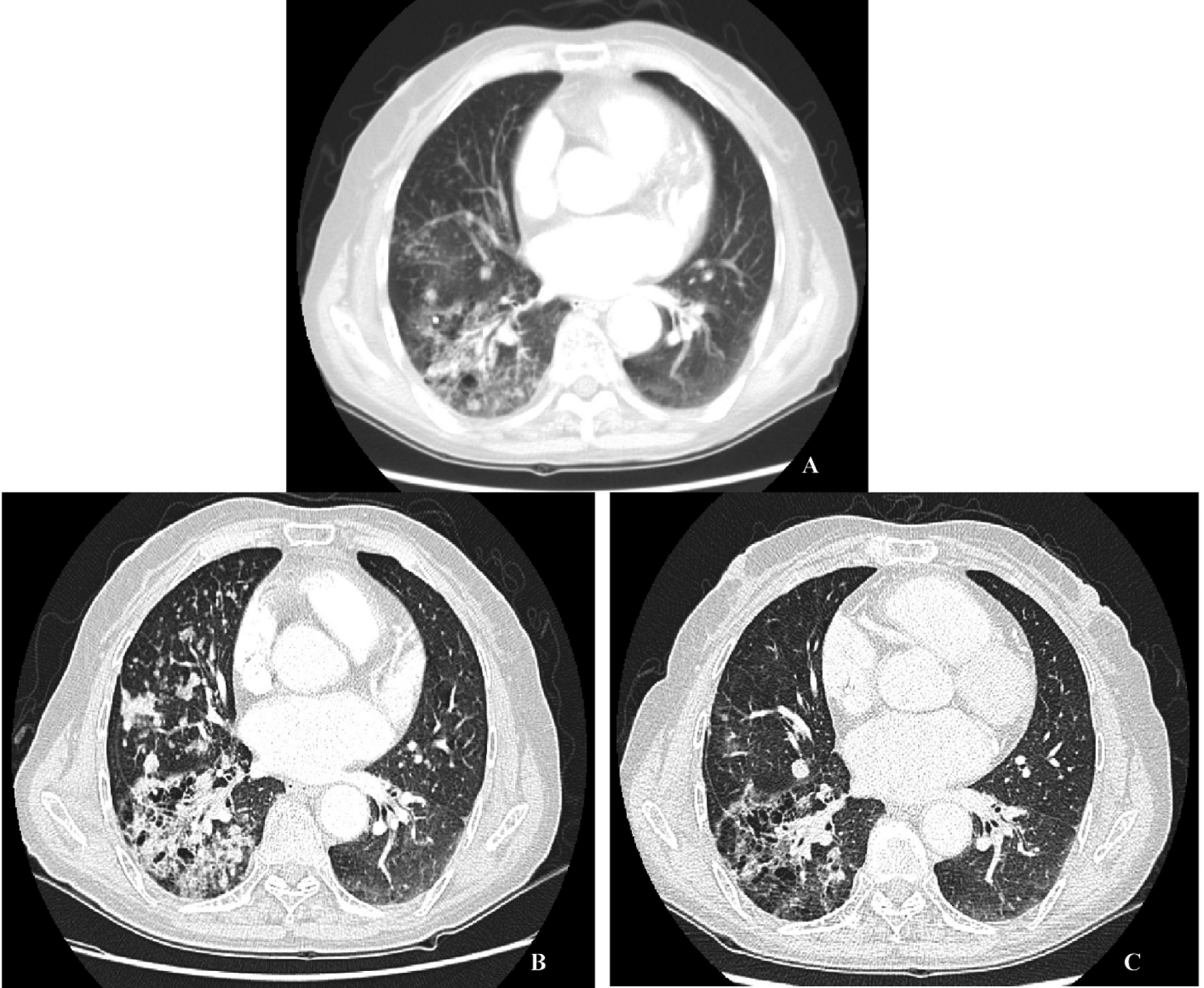
A: Chest CT scan after treatment with 2 courses of pemetrexed, more aggravated consolidative mass in RLL and more enlarged metastatic nodules in RML and RLL. B: Chest CT scan after treatment with 4 courses of gemcitabine/vinorelbine, more aggravated consolidative mass in RLL and more enlarged metastatic nodules in both lungs. C: Chest CT scan 2 months after re-treatment with 250 mg gefitinib, slightly decreased consolidative mass in RLL and markedly decreased metastatic nodules in both lungs.

A 72-years-old nonsmoker woman with fever, chronic cough, purulent sputum, and dyspnea, who had previously taken medication for bronchial asthma for 16 years, was admitted because chest X-ray showed a 5 cm-sized mass-like consolidation in the right lower lung field. Chest computed tomography also noted pneumonic infiltrations in the middle lobe and right lower lobe of the right lung. We started intravenous antibiotics upon diagnosis as possible pneumonia. Bronchial washing was performed because we could not exclude malignancy, such as invasive adenocarcinoma. Washing cytology was negative for malignancy. Although symptoms were improved with antibiotics, a follow-up chest X-ray was not improved. Furthermore, chest CT scans after 4 months showed aggravated infiltrations and newly developed nodules in both of the upper lung fields (Fig. 1A). Percutaneous transthoracic needle aspiration was performed on the patient. Finally, lepidic predominant adenocarcinoma was diagnosed (Fig. 2). After this diagnosis, the patient received the three courses of first-line chemotherapy with docetaxel plus carboplatin as stage IV (T4N0M1) with lung-to-lung metastasis. However, the treatment response was progressive disease (Fig. 1B). Thus, the patient began to take gefitinib at 250 mg/day because EGFR gene analysis in this patient showed a deletion mutation in exon 19 and point mutation L858R in exon 21 by direct sequence method (Fig. 3). A remarkable tumor regression was found as nearly complete response was achieved after 2 months (Fig. 1C). The tumor size of right middle lobe and lower lobe was decreasing, and the metastatic nodules of both upper lobes had nearly disappeared. This response lasted for almost 15 months (Fig. 1D). Although gefitinib was discontinued because the disease had progressed, the patient with a good performance status received 2 courses of pemetrexed and 4 sequential courses of gemcitabine/vinorelbine chemotherapy (Fig. 4A, B). Despite continued chemotherapy, the patient showed radiographic progression. Thus, we commenced re-treatment with gefitinib. Two months later, a partial response had been achieved at the primary tumor and metastatic lesions (Fig. 4C). The response continued even after 4 months. Even after that, the repeated re-administration of gefitinib after 3 to 4 months had elapsed was shown stable disease. The patient was still alive 5 years after she was initially diagnosed with metastatic invasive adenocarcinoma.

## Discussion

We found that a lung cancer patient with lepidic predominant adenocarinoma who had disease progression after a remarkable initial response to gefitinib was again sensitive to re-administration of gefitinib after a certain time had elapsed since the previous gefitinib treatment. So far, there are several patients with adenocarcinoma who, after having experienced disease progression after completely responding to gefitinib, have responded to re-treatment with gefitinib following temporary cessation of gefitinib [3-6]. To our knowledge, it is believed that this is the case report showing a remarkable effect of gefitinib re-treatment in a patient with metastatic invasive adenocarinoma who had experienced favorable results from the initial treatment with gefitinib.

Although an international core panel of experts in IASLC/ATS/ERS recently argued that “lepidic predominant adenocarcinoma (LPA)” or “invasive mucinous adenocarcinoma” should be used instead of the term bronchioloalveolar carcinoma (BAC) [7], the term of BAC could be reserved for those tumors meeting the 1999/2004 criteria set forth by the World Health Organization, which is a form of adenocarcinoma with unique clinical, radiological and epidemiological features [8]. As in our case, LPA with diffuse parenchymal disease is often mistaken for pneumonia or pneumonitis. Although patients may be cured after surgical resection of focal LPA, there is no optimal established therapy for multilobar or recurrent disease. Some clinical trials have been performed specifically with patients with LPA, based on the notion that these tumors may be less sensitive to standard chemotherapy and that the biology of LPA differs from that of other adenocarcinomas [9]. Recently, it has been reported that mutations in the tyrosine kinase domain of the EGFR are associated with the sensitivity of non-small cell lung cancer to gefitinib [10]. Treatment with EGFR tyrosine kinase inhibitors (TKI) gefitinib has led to prompt and dramatic radiologic and clinical improvement in selected patients with metastatic non-small cell lung cancer. Deletions or amino acid substitutions in exons 18, 19, and 21 were found in tumors sensitive to gefitinib. These mutations were frequently found in females, those with adenocarcinoma histology, those of Asian ethnicity, and those with adenocarcinomas of the bronchioloalveolar subtype who had never smoked. In addition, Cadranel et al [11] demonstrated that gefitinib is suitable as a first-line treatment for advanced adenocarcinoma, particularly in patients with lepidic predominant subtype. Our case showed deletion mutation in exon 19 and point mutation L858R in exon 21 as well as pathologically lepidic predominant adenocarcinoma. Based on current studies, we could explain why EGFR-TKI in this patient had a prompt and dramatic radiologic and clinical improvement.

Unfortunately, even in gefitinib responders, disease progression occurs and is inevitable. Some reports present that despite initial responses, patients eventually progress by unknown mechanisms of “acquired” resistance, a secondary mutation in exon 20, which leads to substitution of methionine for threonine at position 790 (T790M) in the kinase domain [12].

More than 4 months after the discontinuation of the initial gefitinib treatment, the patient was retreated with gefitinib due to further progression of the disease. The partial response lasted for 4 months. Re-treatment with gefitinib is known to be effective. So far, little is known about the mechanism of resensitization to gefitinib. Kurata et al [6] suggested three possible explanations for the resensitization in some patients with adenocarcinomas who develop recurrence after successful treatment with gefitinib. First, resistance to gefitinib may naturally change over time. Secondly, the proportion of sensitive or resistant cells might have been modified by chemotherapy after the first treatment with gefitinib. Thirdly, treatment with cytotoxic chemotherapy produces genetic changes in EGFR or other unknown associated genes that regulate resistance to gefitinib.

In conclusion, we investigated a lung cancer patient with lepidic predominat adenocarcinoma who had been successfully controlled with an initial treatment with gefitinib for more than 15 months, who then relapsed and was retreated with gefitinib. The partial response of the patient continued for more than 4 months with re-treatment with gefitinib monotherapy. Further examinations are warranted to clarify the mechanisms of adenocarcinoma sensitivity and resistance to gefitinib.
